# The risk of major bleeding event in patients with chronic kidney disease on pentoxifylline treatment

**DOI:** 10.1038/s41598-021-92753-4

**Published:** 2021-06-29

**Authors:** Jing-Hung Fang, Yi-Chen Chen, Chung-Han Ho, Jui-Yi Chen, Chung-Hsi Hsing, Fu-Wen Liang, Chia-Chun Wu

**Affiliations:** 1 Department of Nephrology, Chi Mei Medical Centre, 901 Zhonghua Road, Yongkang District, Tainan City, 710 Taiwan; 2 Department of Medical Research, Chi Mei Medical Centre, Tainan City, Taiwan; 3 Department of Anaesthesiology, Chi Mei Medical Centre, Tainan City, Taiwan; 4 Department of Public Health, Kaohsiung Medical University, College of Health Sciences, Kaohsiung, Taiwan; 5 Department of Medical Research, Kaohsiung Medical University Hospital, Kaohsiung, Taiwan; 6 Department of Pharmacy, Chia Nan University of Pharmacy and Science, Tainan City, Taiwan

**Keywords:** Drug discovery, Nephrology

## Abstract

Patients with chronic kidney diseases (CKD) are often treated with antiplatelets due to aberrant haemostasis. This study aimed to evaluate the bleeding risk with CKD patients undergoing pentoxifylline (PTX) treatment with/without aspirin. In this retrospective study, we used Taiwan’s National Health Insurance Research Database to identify PTX treated CKD patients. Patients undergoing PTX treatment after CKD diagnosis were PTX group. A 1:4 age, sex and aspirin used condition matched CKD patients non-using PTX were identified as controls. The outcome was major bleeding event (MBE: intracranial haemorrhage (ICH) and gastrointestinal tract bleeding) during 2-year follow-up period. Risk factors were estimated using Cox regression for overall and stratified analysis. The PTX group had higher MBE risk than controls (hazard ratio (HR) 1.19; 95% confidence interval (CI) 0.94–1.50). In stratified analysis, hyperlipidaemia was a significant risk factor (HR: 1.42; 95% CI 1.01–2.01) of MBE. A daily PTX dose larger than 800 mg, females, non-regular aspirin usage, and ischaemic stroke were risk factors for MBE in PTX group. When prescribing PTX in CKD patients, bleeding should be closely monitored, especially in those with daily dose more than 800 mg, aspirin users, and with a history of ischaemic stroke.

## Introduction

CKD was the 10th leading global cause of death in 2019 and also contribute the death from cardiovascular disease. The current recommended managements of CKD include treat reversible cause of kidney injury, control the progression rate of kidney disease, treat and prevent the complications related to CKD. The current pharmacological agents with strong evidences to control CKD are renin–angiotensin–aldosterone-system inhibitors and sodium glucose cotransporter 2 inhibitors (SGLT2i, but individual variability in drug responses and residual risk of CKD needs to be solved. Candidate new drugs such as selective endothelin A receptor antagonist, non-steroidal mineralcorticoid receptors antagonists are tested in clinical trials^1^.

Pentoxifylline (PTX), a non-selective phosphodiesterase inhibitor, is an old drug that has been used to treat vascular circulation disorders since the 1970s with the contraindications of recent cerebral and retinal bleeding^2^. PTX improves microcirculation by reducing the viscosity of red blood cells and inhibiting the platelet aggregation through elevation of platelet cyclic adenosine monophosphate level^3^. The only approved indication of PTX by the United States Food and Drug Administration (US FDA) and the United Kingdom National Institute for Health and Care Excellence (UK NICE) is for treating peripheral vascular disease^4,5^. However, PTX is also found to have anti-inflammatory and anti-fibrotic effects and is off-label used to treat different disorders such as severe alcoholic liver disease, non-alcoholic fatty liver disease, peripartum cardiomyopathy, and chronic kidney diseases (CKD).

Previous studies have tried to elucidate the renal protective role of PTX in CKD. Recently, two meta-analysis studies reported the efficacy of PTX on renal outcomes in CKD patients. Leporini et al.^6^ showed that PTX could reduce proteinuria which was more evident in patients with type 1 diabetes mellitus (DM) and improve the renal function especially in patients with more advanced CKD. Lui et al.^7^ reported that PTX combined with renin-angiotensin blockade could reduce proteinuria and slow down the decline of renal function in the patients with CKD stages 3–5. Although studies on hard renal outcomes of PTX are few, it is still suggested because PTX is relatively safe and cheap; with the most common side effect of gastrointestinal (GI) upset.

Patients with CKD carry a higher risk of bleeding due to platelet dysfunction and anaemia, especial in those with albuminuria^8–10^. The mechanism of platelet dysfunction in CKD patients was caused by uraemia toxins, which interfering the release of platelet alpha granules, platelet adhesion, aggregation, and platelet- vessel wall interaction^11^. On the other hand, CKD itself is an independent risk factor for cardiovascular events^12^ and Jardine et al. demonstrated that the benefit of cardiovascular risk reduction of antiplatelet agents outweighed the risk of major bleeding in CKD patients^13^. Aspirin is the most common antiplatelet agent in the prevention of cardiovascular disease. It inhibits platelet aggregation by inactivating cyclooxygenase to decrease thromboxane A2 formation^14^ and by enhancing fibrinolysis via acetylating fibrinogen^15^. The insufficient antiplatelet effects of aspirin was observed in CKD patients and related to the severity of CKD^16^. In addition, the PTX metabolite M5 that contribute to the hemorheological effects accumulates when the renal function is severely impaired^17^. Under such complicated interference from these conditions, the risk of bleeding due to PTX alone or in combination with aspirin in CKD patients is our concern. Thus, the present study aimed to elucidate this bleeding risk in CKD patients.

## Methods

### Data source

This nationwide population‐based cohort study was conducted using the National Health Insurance Research Database (NHIRD) from the Taiwan’s national health insurance (NHI) program. The NHI program included the primary claims and registration data covering almost 99% population in Taiwan. For providing adequate research data, one million patients enrolled in 2000 were randomly sampled from the beneficiaries of the NHI program to build the Longitudinal Health Insurance Database 2000 (LHID2000). The personal information, prescriptions, covered costs, and the diagnoses and procedure codes based on the International Classification of Diseases, Ninth Revision, Clinical Modification (ICD‐9‐CM) codes were included in LHID2000. The requirement for informed consent was waived due to de-identifiable personal information in LHID2000.

### Ethical statement

The study protocol was reviewed and approved by the Institutional Review Board (IRB) at Chi Mei Medical Center (IRB number: 10703-E01). All methods were performed in accordance with the ethical guidelines of the Declaration of Helsinki. Informed consent from the participants was waived by the IRB because the NHIRD contains anonymized information only. The waiver did not affect the rights and welfare of the participants.

### Study population

**Figure 1:**
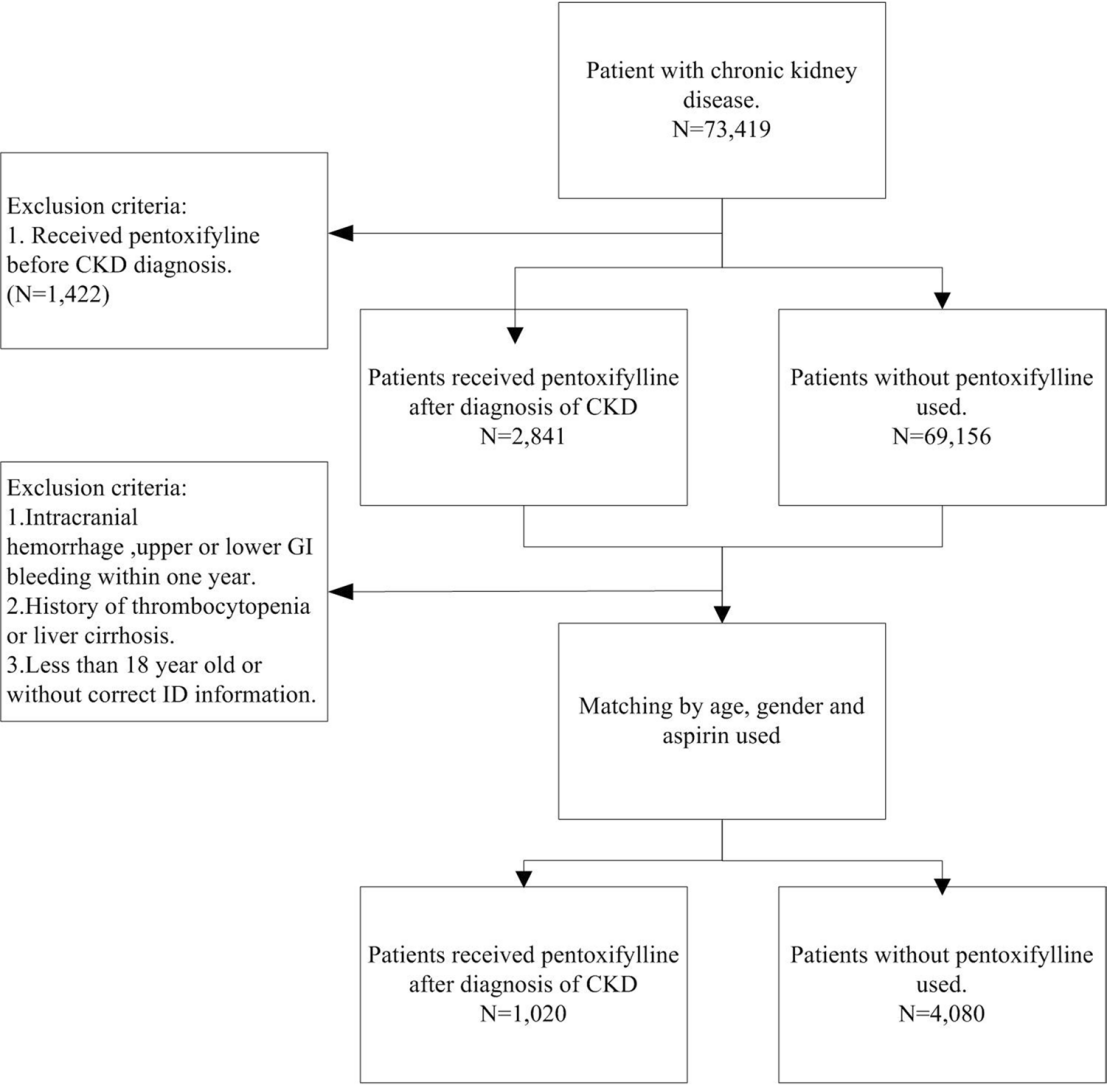
Flowchart of patient population.

Figure 1 represents the flowchart of the study subjects’ selection. CKD patients were selected from LHID2000 based on the ICD-9-CM: 581, 585, 586, 588, 588.8, and 588.9. According to the records of prescriptions, CKD patients using PTX were classified as the case group. The initial PTX record was set as the index date. CKD patients without the records of using PTX were classified as controls, and four controls were matched to each case by diagnosis year of CKD, age, gender, and usage of aspirin. Considering the potential confounding effects, the excluding criteria included CKD patients using PTX before the diagnosis of CKD, bleeding before the index date, and patients with a history of thrombocytopenia (ICD-9-CM: 287.3, 287.4, 287.5) or liver cirrhosis (ICD-9-CM: 571.2, 571.21–571.25, 571.5, 571.51–571.55) before the index date.

### Covariates and outcomes

The covariates in this study included age, gender, usage of aspirin, and comorbidities. The usage of aspirin was categorised as none, non-regular, and regular according to the records of prescriptions. Aspirin prescribed for longer than 3 months without interruption was defined as regular. The comorbidities were defined based on the records of diagnosis within 1 year before patients received PTX, namely DM (ICD-9-CM: 250), Hypertension (ICD-9-CM: 401–405), Hyperlipidaemia (ICD-9-CM: 272), ischaemic stoke (ICD-9-CM: 433–438), and coronary artery disease (ICD-9-CM: 410–414). In addition, end-stage renal disease (ESRD) and erythropoietin (EPO) were considered for the severity of CKD because EPO therapy was covered by national insurance only in CKD stage 5.

The major outcomes of this study were major bleeding events (MBE), which include intracranial haemorrhage (ICH) (ICD-9-CM: 430,431, 432.0, 432.1, 432.9) and GI bleeding (ICD-9-CM: 530.82, 531.0, 531.00, 531.01, 531.2, 531.20, 531.21, 531.4, 531.40, 531.41, 531.6, 531.60, 531.61, 532.0, 532.00, 532.01, 532.2, 532.20, 532.21, 532.4, 532.40, 532.41, 532.6, 532.60, 532.61, 533.0, 533.00, 533.01, 533.2, 533.20, 533.21, 533.4, 533.40, 533.41, 533.6, 533.60, 533.61, 534.0, 534.00, 534.01, 534.2, 534.20, 534.21, 534.4, 534.40, 534.41, 534.6 534.60 534.61 535.01, 535.31, 535.41, 535.51, 535.61, 562.02, 562.03, 562.12, 562.13, 578, 578.0, 578.1, 578.9 for upper GI bleeding; 569.3 for lower GI bleeding). Aspirin, the most commonly used antiplatelet agent, was used as a variate in the analysis and other antiplatelet therapies such as Clopidogrel, Ticlopidine, Cilostazol, and Dipyridamole, and anticoagulants like Heparin, Dalteparin, and Enoxaparin which may affect the bleeding risk were also considered as the covariates. All study subjects were followed-up for a maximum of 2 years after index date or until events occurred i.e. date of death or end of study, 31 December 2013.

### Statistical analysis

Categorical variables are expressed as frequencies with percentages, and Pearson’s chi-squared test was used to compare the difference between patients who received PTX and who did not. In addition, the time to MBE was presented as mean with standard deviation, and patients with and without PTX treatment were compared using the Student’s t-test. The incidence risk for MBE was calculated as the number of events during the follow-up period divided by the total subjects’ person-years. The trends of MBE risk between patients with PTX and those without were plotted using Kaplan–Meier approach, and the log-rank test was used to compare the differences. The relative risks of the MBE in the patients who received PTX and who did not receive PTX were estimated using Cox proportional hazards regression to present the hazard ratio (HR) and 95% confidence interval (CI) with the adjustment of age, gender, comorbidities, and other clinical confounding factors. The subgroup analysis by age, gender, aspirin usage, and ESRD also presented the adjusted HRs. Furthermore, the potential risk factors for the MBE among patients who received PTX were also presented. P-value of < 0.05 was considered as statistically significant. All analyses were performed using SAS 9.4 (SAS Institute Inc., Cary, NC, USA). The trend for probability of MBE was plotted using STATA 12.0 (Stata Corp., College Station, TX, USA).

## Results

A total of 73,419 patients diagnosed with CKD were identified. After excluding patients who received PTX before the diagnosis of CKD, finally, 71,997 patients were included. Of these, 2841 patients received PTX treatment and 69,156 patients did not receive PTX treatment after the diagnosis of CKD. After applying the exclusion criteria, 1020 patients treated with PTX were selected as the study group (PTX group) and 1:4 age, gender, and aspirin use condition matched patients were chosen as the control group (Fig. 1). In the PTX group, 55.88% patients were older than 60 years and were males. The proportions of patients with DM, hypertension, hyperlipidaemia, and a history of ischaemic stroke were significantly higher in the PTX (P < 0.0001) whereas the proportion of patients with ESRD was significantly less than the control group (30 vs. 204, P = 0.0049). The PTX group had significantly more patients on DAPT/anticoagulants than the control group (16.47 vs. 10.98%, P < 0.0001).

The rate of death was 4.8% (49/1020) in the PTX group and 5.61% (229/4080) in the control group with no significant difference between the groups. The rate of MBE was significant higher in the PTX as compared to the control group (9.61% vs. 7.5%, P = 0.026). Upper GI tract bleeding contributed the majority of the MBE in the PTX (90.4%) and the control (83.9%) groups, with no difference between the two groups. The ICH rate was not as high as the upper GI tract bleeding, but was significantly increased in the PTX group as compared to the control group (1.57 vs. 0.69%, P = 0.006). The median time to the first MBE from the beginning of the study was 7.21 and 7.93 months in the PTX and control groups, respectively (Table 1).

**Table 1:** Demographic information of patients with chronic kidney disease.

	Pentoxifylline (n = 1020)	Control (n = 4080)	P-value
**Age group (years)**			1.0000
18–59, n (%)	450 (44.12)	1800 (44.12)	
60 and above, n (%)	570 (55.88)	2280 (55.88)	
**Gender**			1.0000
Female, n (%)	450 (44.12)	1800 (44.12)	
Male, n (%)	570 (55.88)	2280 (55.88)	
**Aspirin used**			1.0000
No, n (%)	803 (78.83)	3212 (78.73)	
Non-regular, n (%)	145 (14.22)	580 (14.22)	
Regular, n (%)	72 (7.06)	288 (7.06)	
**Comorbidities**			
DM, n (%)	457 (44.80)	1370 (33.58)	< 0.0001
HTN, n (%)	748 (73.33)	2337 (57.28)	< 0.0001
Hyperlipidaemia, n (%)	449 (44.02)	1398 (34.26)	< 0.0001
Ischaemic stroke, n (%)	188 (18.43)	431 (10.56)	< 0.0001
ESRD, n (%)	30 (2.94)	204 (5.00)	0.0049
CKD stage 5, n (%)	6 (0.59)	52 (1.27)	0.0645
DAPT/anticoagulants n (%)	168 (16.47)	448 (10.98)	< 0.0001
**Pentoxifylline**			
< 800 mg/day, n (%)	505 (49.51)	–	
≥ 800 mg/day, n (%)	515 (50.49)	–	
Mortality, n (%)	49 (4.80)	229 (5.61)	0.3088
**MBE, n (%)**	98 (9.61)	306 (7.50)	0.0258
Intracranial haemorrhage, n (%)	16 (1.57)	28 (0.69)	0.0064
Upper GI bleeding, n (%)	79 (7.75)	262 (6.42)	0.1301
Lower GI bleeding, n (%)	3 (0.29)	17 (0.42)	0.5754
**Time to MBE (months)**			
Mean ± SD	8.51 ± 6.09	8.72 ± 6.65	0.7899
Median(Q1–Q3)	7.21 (3.30–13.20)	7.93 (3.00–13.43)	

CKD, chronic kidney disease; DAPT, dual antiplatelet therapy; DM, diabetes mellitus; ESRD, end-stage renal disease; GI, gastrointestinal; HTN, hypertension; MBE, major bleeding event; SD, standard deviation.

**Figure 2:**
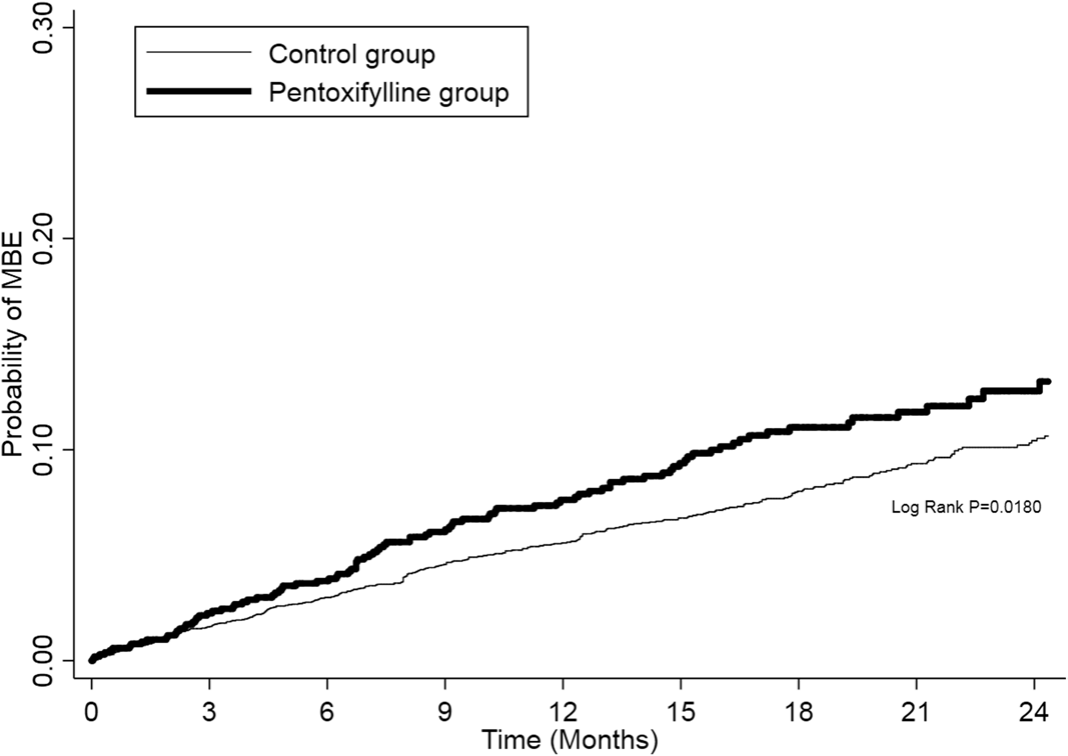
Probability of major bleeding event between pentoxifylline patients and controls.

The PTX group had a significant higher probability of MBE than the control group (Log-rank test for trend, p = 0.018) (Fig. 2). The risk of MBE was significantly increased in the PTX group when compared to the control group (crude HR(CHR) 1.27, 95% CI 1.01–1.6). In the subgroup analysis, female patients, non-dialysis dependent CKD patients, and patients with hyperlipidaemia in the PTX group had 1.41 times (95% CI 1.02–1.95), 1.32 times (95% CI 1.05–1.67), and 1.41 times (95% CI 1.00–1.97) increased risk of the MBE respectively. After adjusting the covariables shown in Table 1, hyperlipidaemia remained significant with an adjusted HR of 1.41 (95% CI 1.01–2.01). A higher rate of MBE (more than 100 events per-1000 person-year) was noted in patients who had received combined treatment of aspirin and PTX and who had a history of ischaemic stroke either in the PTX or control group (Table 2).

**Table 2:** The overall and subgroup analysis of haemorrhage or bleeding risk in patients with chronic kidney disease treated with and without pentoxifylline.

Variable	Pentoxifylline (n = 1020)			Control (n = 4080)			CHR (95% CI)	AHR^a^ (95% CI)
	Case (%)	PY	Rate	Case (%)	PY	Rate		
Overall analysis	98 (9.61)	1279.22	76.62	306 (7.50)	5079.78	60.24	1.27 (1.01–1.60)	1.19 (0.94–1.50)
Subgroup analysis								
**Age group**								
18–59	34 (7.56)	579.35	58.69	99 (5.50)	2332.67	42.44	1.38 (0.94–2.04)	1.28 (0.85–1.92)
60 and above	64 (11.23)	699.87	91.45	207 (9.08)	2747.12	75.35	1.21 (0.92–1.61)	1.14 (0.86–1.52)
**Gender**								
Female	49 (10.89)	554.81	88.32	139 (7.72)	2219.15	62.64	1.41 (1.02–1.95)	1.34 (0.96–1.86)
Male	49 (8.60)	724.41	67.64	167 (7.32)	2860.63	58.38	1.16 (0.84–1.59)	1.07 (0.77–1.48)
**Aspirin used**								
No	65 (8.09)	1031.27	63.03	214 (6.66)	4072.23	52.55	1.20 (0.91–1.58)	1.08 (0.81–1.44)
Non-regular	23 (15.86)	159.50	144.21	62 (10.69)	651.63	95.15	1.52 (0.94–2.45)	1.43 (0.87–2.35)
Regular	10 (13.89)	88.46	113.05	30 (10.42)	355.93	84.29	1.34 (0.66–2.75)	1.32 (0.63–2.77)
**ESRD**								
No	95 (9.60)	1243.18	76.42	280 (7.22)	4842.25	57.82	1.32 (1.05–1.67)	1.21 (0.95–1.53)
Yes	3 (10.00)	36.04	83.24	26 (12.75)	237.53	109.46	0.78 (0.24–2.56)	0.70 (0.21–2.39)
DM	52 (11.38)	574.90	90.45	120 (8.76)	1635.22	73.38	1.24 (0.89–1.71)	1.23 (0.88–1.70)
HTN	77 (10.29)	937.20	82.16	195 (8.34)	2836.00	68.76	1.20 (0.92–1.56)	1.21 (0.92–1.58)
Hyperlipidemia	38 (10.69)	549.44	69.16	110 (7.87)	1775.45	61.96	1.41 (1.00–1.97)	1.42 (1.01–2.01)
Ischaemic stroke	31 (16.49)	230.43	134.53	50 (11.60)	485.50	102.98	1.32 (0.84–2.06)	1.33 (0.84–2.09)
CAD	28 (12.44)	280.69	99.75	78 (10.57)	870.02	89.65	1.12 (0.73–1.72)	1.12 (0.72–1.75)

AHR, adjusted hazard ratio; CAD, coronary artery disease; CHR, crude hazard ratio; DM, diabetes mellitus; ESRD, end-stage renal disease; HTN, hypertension.

^a^Covariates adjusted by age groups, gender, group of aspirin used, DM, HTN, hyperlipidaemia, ischaemic stoke, ESRD, CKD stage 5 and dual antiplatelet therapy or anticoagulants^.^ PY:1000 person year.

Since the PTX group had a significant increased risk of MBE than the controls, we further conducted an analysis to identify the factors contributing to this risk among patients under PTX treatment. The analytic results showed that a daily PTX dosage more than 800 mg would cause a 73% increased risk of MBE than the daily dose less than 800 mg (95% CI 1.14–2.62); non-regular aspirin prescription would increase the risk of MBE by 80% than no aspirin prescription (95% CI 1.06–3.04), and a past history of ischaemia stoke would increase a 76% of risk of MBE (95% CI 1.12–2.75) (Table 3). Although the rate of ICH is higher in the PTX group, the demographic characteristics of patients with ICH were not different between the PTX and the control groups (Table 4).

**Table 3:** Risk factor of major bleeding event in patients with chronic kidney disease receiving pentoxifylline.

	CHR (95% CI)	AHR (95% CI)	P-value
**Pentoxifylline**			
< 800 mg/day	Ref.	Ref.	
≥ 800 mg/day	1.68 (1.11–2.52)	1.73 (1.14–2.62)	0.0095
**Age group**			
18–59	Ref.	Ref.	
60 ≥	1.55 (1.02–2.35)	1.29 (0.83–2.01)	0.2545
**Gender**			
Female	Ref.	Ref.	
Male	0.77 (0.5–1.15)	0.68 (0.46–1.02)	0.0599
**Aspirin used**			
No	Ref.	Ref.	
Non-regular	2.24 (1.39–3.61)	1.80 (1.06–3.04)	0.0291
Regular	1.78 (0.91–3.46)	1.42 (0.70–2.85)	0.3319
**Comorbidities (compared with no such comorbidity)**			
DM	1.38 (0.93–2.06)	1.17 (0.77–1.77)	0.4518
HTN	1.34 (0.83–2.17)	1.13 (0.69–1.85)	0.6395
Hyperlipidaemia	1.27 (0.86–1.89)	1.35 (0.89–2.04)	0.1552
Ischemia stroke	2.10 (1.37–3.21)	1.76 (1.12–2.75)	0.0140
CAD	1.43 (0.92–2.21)	1.04 (0.65–1.68)	0.8715
ESRD	1.08 (0.34–3.40)	1.37 (0.42–4.44)	0.6011
DAPT/anticoagulants	1.48 (0.90–2.44)	1.23 (0.73–2.06)	0.4322

AHR, adjusted hazard ratio; CAD, coronary artery disease; CHR, crude hazard ratio; DAPT, dual antiplatelet therapy; DM, diabetes mellitus; ESRD, end-stage renal disease; HTN, hypertension.

**Table 4:** Demographic information of patients with intracranial haemorrhage.

	Pentoxifylline (n = 16)	Control (n = 28)	P-value
**Age group (years)**			0.7237
18–59, n (%)	3 (18.75)	7 (25.00)	
60 and above, n (%)	13 (81.25)	21 (75.00)	
**Gender**			0.8195
Female, n (%)	8 (50.00)	13 (46.43)	
Male, n (%)	8 (50.00)	15 (53.57)	
**Aspirin used**			0.7625
No, n (%)	12 (75.00)	17 (60.71)	
Non-regular, n (%)	3 (18.75)	9 (32.14)	
Regular, n (%)	1 (6.25)	2 (7.14)	
**Comorbidities**			
DM, n (%)	8 (50.00)	14 (50.00)	1.0000
HTN, n (%)	13 (81.25)	23 (82.14)	1.0000
Hyperlipidaemia, n (%)	7 (43.75)	8 (28.57)	0.3069
Ischaemic stroke, n (%)	4 (25.00)	9 (32.14)	0.7385
CAD, n (%)	4 (25.00)	7 (25.00)	1.0000
ESRD, n (%)	0 (0.00)	4 (14.29)	0.2797
EPO, n (%)	–	–	
DAPT/anticoagulant, n (%)	4 (25.00)	6 (21.43)	1.0000
**Pentoxifylline**			
< 800 mg/day, n (%)	8 (50.00)	–	
≥ 800 mg/day, n (%)	8 (50.00)	–	
Mortality, n (%)	5 (31.25)	10 (35.71)	0.7638

CAD, coronary artery disease; DAPT, dual antiplatelet therapy; DM, diabetes mellitus; ESRD, end-stage renal disease; EPO, erythropoietin; HTN, hypertension.

## Discussion

In spite of RAASi and SGLT2i have strong evidence to control CKD progression, PTX is still considered for renoprotection because of its safety and low cost for the residual risk. Patients with CKD have abnormal haemostasis, presented by both bleeding tendency and hypercoagulability which contributes to a higher risk of both bleeding and cardiovascular diseases. To prevent cardiovascular diseases, antiplatelet agent treatment is common in CKD patients. Thus, in cases where PTX treatment is additionally given to the CKD patients already undergoing antiplatelet treatment, it becomes necessary to evaluate the safety of the combined treatment. Our data showed that the CKD patients who underwent PTX treatment had a significantly higher rate of the MBE than the matched patients without PTX treatment. The reason of higher risk of MBE in the PTX group may attribute to higher percentage of hypertension and DAPT/anticoagulant that vulnerable to ICH and the function on hemorheology of PTX itself. Most MBE developed within 1 year of the PTX treatment.

The results also showed female sex as a risk factor for MBE in patients undergoing PTX treatment. Otahbachi et al. studied the gender difference in platelet aggregation in healthy individuals of different ethnicity and the data showed that the women had a greater platelet aggregation in response to the test agonists^18^. Berger et al. conducted a sex-specific meta-analysis of aspirin treatment for CVD prevention and found that aspirin could reduce the risk of ischaemic stroke in women and that of myocardial infarction in men, with an increased risk of major bleeding without significant gender differences^19^. In another sex-specific meta-analysis on the efficiency and safety of clopidogrel published by the same author, no significant difference in efficiency but a significant increased bleeding event in women receiving a combination of clopidogrel and aspirin was observed^20^. Mehran et al. showed that female sex was a predictor of major bleeding in acute coronary syndrome^21^. According to these reports, the gender difference in platelet reactivity and response to antiplatelet agents may explain our findings that the female sex is a risk factor for bleeding related to PTX treatment.

Combination of aspirin and PTX would increase the bleeding risk than treatment with PTX alone is a result of generalized expectancy. We matched the factor of aspirin user trying to decrease the bias and clarify its role in the MBE. The subgroup analysis showed add-on PTX was not an insignificant risk factor of MBE in patients treated with aspirin. However, in the analysis of patients treated PTX, data revealed that the patients who had irregular aspirin prescription carried a significant higher risk for MBE. We could not identify the true reason for the increased bleeding risk with irregular aspirin prescription but might be explained by non-adherence due to medical conditions or non-compliance of the patients. The small number of MBE (n = 3) in the ESRD patients might cause misinterpretation thus, we did not discuss the result of the lower HR in this subgroup.

Hyperlipidaemia is an important risk factor of atherosclerosis and is considered to be associated with hypercoagulation^22^. Statin is a major drug to treat hyperlipidaemia (hypercholesterolaemia) and to protect patients from cardiovascular diseases. Besides the lipid-lowering effect, statins were found to have an anticoagulant effect^23^. Statin alone or in combination with other antiplatelets/anti-coagulants might increase the risk of bleeding including GI tract and ICH, as reported in some studies^24–27^. However, other studies reported the contradictory results^28–30^. In a recent review that investigated this issue in both randomized controlled trials and observational studies, no significant evidence to support the role of statin therapy in the increased risk of ICH was found^31^. Based on a retrospective study of 10-year trends in new statin users in Taiwan, approximately 76% of new statin users had a diagnosis of hyperlipidaemia^32^. We hypothesize that the finding of hyperlipidaemia as a risk factor for MBE under PTX treatment might be secondary to the statin use.

This is the first study to clarify the bleeding risks in CKD patients treated with PTX, which might be helpful in patient care and safety during clinical practice. However, several limitations of this study should be acknowledged. First, we did not have detailed personal information, such as lifestyle, blood pressure control, and drug compliance. Secondly, the detailed laboratory information and clinical symptoms for our study subjects were not available. Therefore, future research is needed to further validate our findings on bleeding risk among CKD patients treated with PTX. Third, some subgroups presented the rare events. The interpretation for these subgroups should be carefully as more sample sizes and relative information are required. Finally, the potential misclassification bias may be possible in this secondary database due to the definition of CKD, comorbidities, and bleeding based on the ICD-9-CM. However, the previous study had validated the NHIRD with higher positive predictive value^33^. Thus, we think the misclassification bias was negligible.

In conclusion, although the meta-analysis studies showed some benefit of PTX in CKD patients^6,7^, we suggest that the risk/benefit ratio should be considered before prescription, especially in patients with hyperlipidaemia which emerged as the most prominent risk factor of MBE in our study. When treating CKD patients with PTX, a dose of 400 mg per day might be safer than the dose of more than 800 mg per day. Furthermore, patients should be cautioned for the potential bleeding event in the first year of PTX use especially when patients have a history of ischaemic stroke or irregular aspirin use.

## References

[1] Provenzano M (2021). Selective endothelin A receptor antagonism in patients with proteinuric chronic kidney disease. Expert Opin. Investig. Drugs..

[2] Kellner H (1976). Treatment of chronic arterial circulatory disorders. Double blind trial with Trental 400 (author's transl). MMW Munch Med. Wochenschr..

[3] Weithmann KU (1983). Reduced platelet aggregation by effects of pentoxifylline on vascular prostacyclin isomerase and platelet cyclic AMP. Gen. Pharmacol..

[4] US Food & Drug Administration: FDA Approved Drug Products. https://www.accessdata.fda.gov/drugsatfda_docs/label/2012/018631s039lbl.pdf.

[5] United Kingdom National Institute for Health and Care Excellence. Prescribing and Technical Information.: http://bnf.nice.org.uk/drug/pentoxifylline.html.

[6] Leporini C (2016). Effect of pentoxifylline on renal outcomes in chronic kidney disease patients: A systematic review and meta-analysis. Pharmacol. Res..

[7] Liu D (2017). Pentoxifylline plus ACEIs/ARBs for proteinuria and kidney function in chronic kidney disease: A meta-analysis. J. Int. Med. Res..

[8] Ferguson JH, Lewis JH, Zucker MB (1956). Bleeding tendency in uremia. Blood.

[9] Ocak G (2018). Chronic kidney disease and bleeding risk in patients at high cardiovascular risk: A cohort study. J. Thromb. Haemost..

[10] Acedillo RR (2013). The risk of perioperative bleeding in patients with chronic kidney disease: A systematic review and meta-analysis. Ann. Surg..

[11] Lutz J, Menke J, Sollinger D, Schinzel H, Thurmel K (2014). Haemostasis in chronic kidney disease. Nephrol. Dial Transplant..

[12] Anavekar NS, Pfeffer MA (2004). Cardiovascular risk in chronic kidney disease. Kidney Int. Suppl..

[13] Jardine MJ (2010). Aspirin is beneficial in hypertensive patients with chronic kidney disease: A post-hoc subgroup analysis of a randomized controlled trial. J. Am. Coll. Cardiol..

[14] Awtry EH, Loscalzo J (2000). Aspirin. Circulation.

[15] Bjornsson TD, Schneider DE, Berger H (1989). Aspirin acetylates fibrinogen and enhances fibrinolysis. Fibrinolytic effect is independent of changes in plasminogen activator levels. J. Pharmacol. Exp. Ther..

[16] Polzin A (2016). Antiplatelet effects of aspirin in chronic kidney disease patients. J. Thromb. Haemost..

[17] Paap CM (1996). Multiple-dose pharmacokinetics of pentoxifylline and its metabolites during renal insufficiency. Ann Pharmacother..

[18] Otahbachi M (2010). Gender differences in platelet aggregation in healthy individuals. J. Thromb. Thrombolysis.

[19] Berger JS (2006). Aspirin for the primary prevention of cardiovascular events in women and men: A sex-specific meta-analysis of randomized controlled trials. JAMA.

[20] Berger JS (2009). The relative efficacy and safety of clopidogrel in women and men a sex-specific collaborative meta-analysis. J. Am. Coll. Cardiol..

[21] Mehran R (2010). A risk score to predict bleeding in patients with acute coronary syndromes. J. Am. Coll. Cardiol..

[22] Kim JA, Kim JE, Song SH, Kim HK (2015). Influence of blood lipids on global coagulation test results. Ann. Lab. Med..

[23] Undas A, Brummel-Ziedins KE, Mann KG (2014). Anticoagulant effects of statins and their clinical implications. Thromb. Haemost..

[24] Martinez AI, Freeman PR, Moga DC (2019). Statin use and gastrointestinal hemorrhage: A large retrospective cohort study. Am. J. Cardiovasc. Drugs.

[25] Hackam DG (2012). Statins and intracerebral hemorrhage: A retrospective cohort study. Arch. Neurol..

[26] Shin D (2016). Comparison of the risk of gastrointestinal bleeding among different statin exposures with concomitant administration of warfarin: Electronic health record-based retrospective cohort study. PLoS ONE.

[27] Antoniou T (2017). Association between statin use and ischemic stroke or major hemorrhage in patients taking dabigatran for atrial fibrillation. CMAJ.

[28] Ho BL (2019). Statins and the risk of bleeding in patients taking dabigatran. Acta Neurol. Scand..

[29] Jung M, Lee S (2019). Effects of statin therapy on the risk of intracerebral hemorrhage in Korean patients with hyperlipidemia. Pharmacotherapy.

[30] Badillo R, Schmidt R, Mortensen EM, Frei CR, Mansi I (2015). Statin therapy and gastrointestinal hemorrhage: A retrospective cohort study with propensity score-matching. Pharmacoepidemiol. Drug Saf..

[31] Newman CB (2019). Statin safety and associated adverse events: A scientific statement from the American Heart Association. Arterioscler. Thromb. Vasc. Biol..

[32] Hsieh HC, Hsu JC, Lu CY (2017). 10-year trends in statin utilization in Taiwan: A retrospective study using Taiwan's National Health Insurance Research Database. BMJ Open.

[33] Hsieh CY (2019). Taiwan's National Health Insurance Research Database: Past and future. Clin. Epidemiol..

